# Polyphenols in Alzheimer’s Disease and in the Gut–Brain Axis

**DOI:** 10.3390/microorganisms8020199

**Published:** 2020-01-31

**Authors:** V. Prakash Reddy, Puspa Aryal, Sara Robinson, Raheemat Rafiu, Mark Obrenovich, George Perry

**Affiliations:** 1 Department of Chemistry, Missouri University of Science and Technology, Rolla, MO 65409, USA; pa8qd@umsystem.edu (P.A.); ssrhcd@mst.edu (S.R.); rbrvkf@mst.edu (R.R.); 2 Department of Veteran’s Affairs Medical Center, Louis Stokes Cleveland VA Medical Center, Cleveland, OH 44106, USA; dr.marke.obrenovich@gmail.com; 3 Department of Biology, University of Texas at San Antonio, San Antonio, TX 78249, USA; George.Perry@utsa.edu

**Keywords:** gut–brain axis, Alzheimer’s disease, antioxidants, glycation, AGEs, flavonoids, polyphenols, enterolactone, neuroprotection, reactive oxygen species

## Abstract

Polyphenolic antioxidants, including dietary plant lignans, modulate the gut–brain axis, which involves transformation of these polyphenolic compounds into physiologically active and neuroprotector compounds (called human lignans) through gut bacterial metabolism. These gut bacterial metabolites exert their neuroprotective effects in various neurodegenerative diseases, such as Alzheimer’s disease (AD) and Parkinson’s disease (PD), and also have protective effects against other diseases, such as cardiovascular diseases, cancer, and diabetes. For example, enterolactone and enterodiol, the therapeutically relevant polyphenols, are formed as the secondary gut bacterial metabolites of lignans, the non-flavonoid polyphenolic compounds found in plant-based foods. These compounds are also acetylcholinesterase inhibitors, and thereby have potential applications as therapeutics in AD and other neurological diseases. Polyphenols are also advanced glycation end product (AGE) inhibitors (antiglycating agents), and thereby exert neuroprotective effects in cases of AD. Thus, gut bacterial metabolism of lignans and other dietary polyphenolic compounds results in the formation of neuroprotective polyphenols—some of which have enhanced blood–brain barrier permeability. It is hypothesized that gut bacterial metabolism-derived polyphenols, when combined with the nanoparticle-based blood–brain barrier (BBB)-targeted drug delivery, may prove to be effective therapeutics for various neurological disorders, including traumatic brain injury (TBI), AD, and PD. This mini-review addresses the role of polyphenolic compounds in the gut–brain axis, focusing on AD.

## 1. Introduction

Polyphenolic antioxidant compounds are mostly plant-derived phenolic compounds—some of which may exist as their ether (e.g., ferulic acid) or ester (e.g., epigallocatechin gallate) derivatives. Polyphenolic compounds are a diverse class of phenolic compounds that include flavanoids, such as epigallocatechin, epigallocatechin gallate, and catechin, and flavonoids, such as quercetin, fisetin, and luteolin, as pharmaceutically important compounds [1,2,3,4]. Quercetin and related flavonoids are β-amyloid precursor protein–cleaving enzyme 1 (BACE-1) inhibitors [5]. Flavanoids as well as non-steroidal anti-inflammatory agents (NSAIDS) modulate the nuclear factor-kappa β (NF-κB) signaling pathways, and thereby are potential therapeutic targets for neurodegenerative diseases, such as AD and Parkinson’s disease (PD) [2].

**Figure 1:**
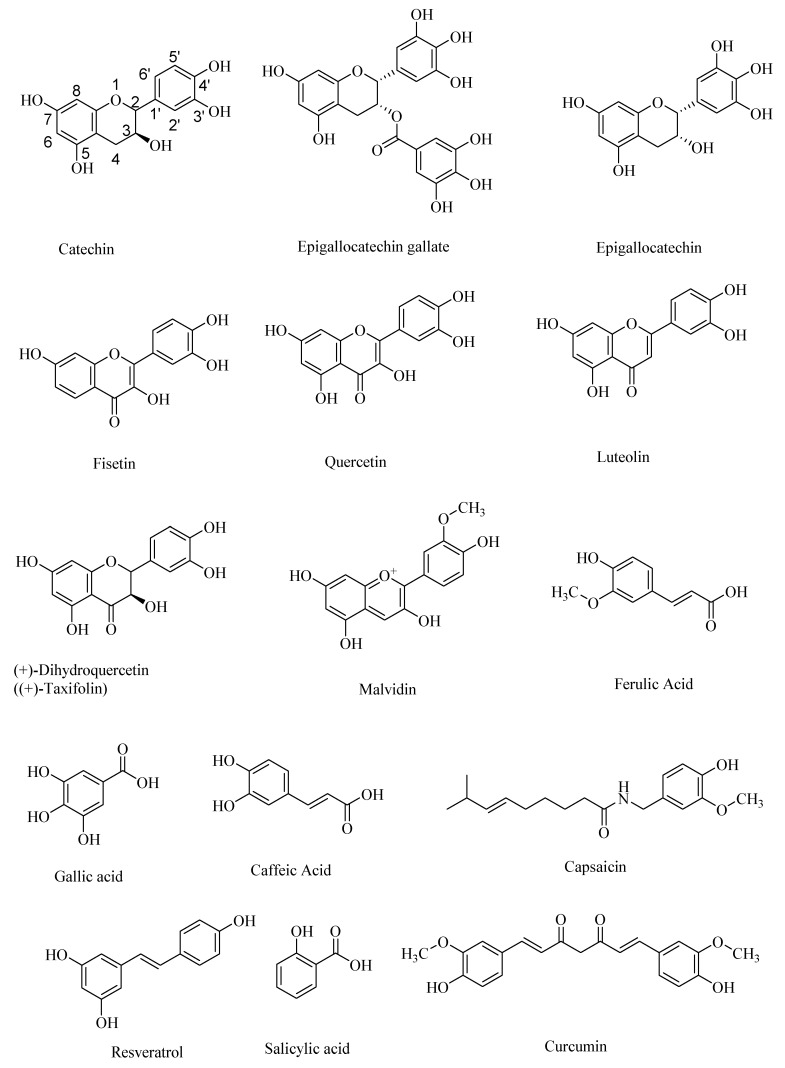
Structures of some of the naturally occurring polyphenolic compounds.

Other naturally occurring polyphenolic compounds include anthocyanidins, such as malvidin, and non-flavanoids/flavonoids, such as ferulic acid, gallic acid, caffeic acid, salicylic acid, and capsaicin (Figure 1). Plants have been the major source of naturally occurring polyphenols that are either synthesized during the normal development of plant tissue or as the defense against pathogens [6,7]. Many polyphenols have various health benefits and constitute the largest quantities of antioxidants in the human diet. With over 8000 known polyphenols occurring naturally, they are one of the most diverse, naturally occurring antioxidants. Polyphenols act as free radical scavengers, and thereby afford protection from chronic diseases, such as Alzheimer’s disease and diabetes, in which free radicals play a major role in the pathogenesis. For example, polyphenolic phytochemicals occurring in various plant sources, including black soybeans, are effective in maintaining human health, especially in preventing cardiovascular diseases, neurological disorders, cancer, and diabetes, although their therapeutic effect on the progress of the Alzheimer’s disease (AD) is debated, perhaps due to the irreversible neuronal damage at the later stages of AD [8]. 

There are many natural sources of polyphenols, including fruits, vegetables, grains, nuts, and seeds [9,10,11,12,13,14]. Some of the most common forms of naturally occurring polyphenols include flavonoids, tannins, phenolic acids, lignans, and stilbene moiety-containing polyphenols, such as resveratrol (Figure 1). Plant-based lignans are metabolized to give the secondary metabolites, also called human lignans—some of which can cross the blood–brain barrier and act as neuroprotectors, in diseases such as AD and Autism Spectrum disorders (ASD) [15]. Dietary polyphenolic compounds modulate the gut microbiota to generate the therapeutically relevant polyphenolic compounds with improved blood–brain barrier permeabilities and, accordingly, the gut–brain axis may afford novel therapeutic routes to treat the devastating neurological disorders. For example, the naturally occurring polyphenolic compound curcumin (Figure 1) protects against AD due to its amyloid-β binding effectiveness and antioxidant effects, although it has limited blood–brain barrier crossing ability. The current working hypothesis for the neuroprotective effect of curcumin is that it reacts indirectly with the central nervous system through its interaction with the gut–microbiota–brain axis [16]. It is possible that the curcumin is transformed into more effective neuroprotective agents through the gut microbial metabolism. Alternatively, because of its indirect effect on the gut microbial metabolic pathways [17], the gut–brain axis is favorably modulated to provide neuroprotection. 

## 2. Polyphenols as Antioxidants

The antioxidant behavior of polyphenols involves various mechanistic pathways, and in some cases, with multistep mechanisms. However, one of the widely accepted mechanisms for the antioxidant behavior of polyphenols is through free radical scavenging. The key structural feature of the radicals formed from the polyphenolic antioxidants is the extended conjugation of the unpaired electron, and thereby enhanced stability. This enables polyphenolic compounds to display antioxidant properties by quenching free radicals, including reactive oxygen species (ROS) and reactive nitrogen species (RNS). The ROS and RNS commonly observed during the free radical-induced oxidations include hydroxyl radical (·OH), superoxide radical anion (O_2_**^–^**), nitric oxide (·NO) and peroxynitrite anion (O=NOO^–^). The ROS and RNS are generated through the metal-ion (e.g., Fe^2+^ and Cu^+^) catalyzed Fenton reactions. The high reactivity and short half-lives of the ROS and RNS, in combination with an imbalance in their formation versus destruction under enhanced oxidative stress, results in the accumulation of excessive amounts of ROS and RNS-derived protein modifications and other reaction products at the sites of inflammation. This excessive accumulation of the ROS and RNS-derived reaction products at the sites of inflammation is a causative factor for various chronic diseases, including AD, traumatic brain injury (TBI), atherosclerosis, and cancer. 

Various assays, such as TEAC (trolox-equivalent antioxidant activity) assay, ORAC (oxygen radical absorbance capacity), ABTS (2, 2′-azino-bis-3-ethylbenzthiazoline-6-sulphonic acid) assay, and DPPH (1, 1-diphenyl-2-picrylhydrazyl) assay have been developed to determine the relative antioxidant ability of polyphenols [18]. However, antioxidant assays based on free radical trapping have had limited success. Without accounting for the function of enzymes in the process, these assays cannot accurately quantify the antioxidant effectiveness of polyphenolic compounds [19]. Furthermore, these antioxidant assays also do not take into account the Fe^2+^ or Cu^+^ ion-binding of polyphenols, which would result in lowering their antioxidant potential [20]. While there are numerous epidemiological studies, such as in vitro and animal testing that show a correlation between dietary intake and decreased risk of multiple chronic diseases, other studies suggest that polyphenol absorption through dietary intake is not an optimal method to treat diseases, because the compounds are, in general, metabolized to give the secondary metabolites, which are structurally distinct compounds, and therefore, by themselves may be antioxidants or may lack antioxidant properties. On the other hand, polyphenolic compounds can exert their therapeutic effectiveness, independent of their antioxidant properties, through their interactions with proteins and phospholipids in the plasma membranes, thereby regulating the signal transduction pathways, and other biochemical pathways, such as DNA methylation and mitochondrial function that afford their beneficial effects [21].

## 3. Polyphenols in Alzheimer’s Disease

Alzheimer’s disease (AD) is a progressive disorder that may lead to the death of neuronal cells and eventually to memory loss. AD contributes to approximately 65% of all dementia cases, and this number is expected to grow substantially [22,23]. In US, there were approximately five million AD cases in 2017, and this number of AD cases is predicted to rise to 16 million by 2050. It is estimated that 1 out of 85 people worldwide would be diagnosed with AD by 2050 [24,25]. Patients with AD experience a slow but gradual decline in memory, thinking, and reasoning skills, with the accompanying dementia that gradually increases in severity, leading eventually to the patient’s death. Currently, medications to treat AD afford only symptomatic relief, and do not treat the underlying cause. While the actual mechanism for the disease remains somewhat unclear, it is generally agreed that the oxidative stress, resulting from the depletion of antioxidants, is a primary characteristic of AD [26]. Other possible causes of AD include oxidative stress-induced neuroinflammation and glutamatergic excitotoxicity.

### 3.1. Oxidative Stress in the Formation of the Aβ Protein Aggregates

Numerous studies suggested that the polyphenols exert their beneficial effects through induction of antioxidant defenses, through attenuation of the protein oxidation, and by lowering the blood pressure [27]. These elevated oxidative stress levels precede other key indicators of the disease, which include the formation of the intercellular aggregates of the amyloid-β peptides and mitochondrial damage. The neurotoxic amyloid-β1–42 peptides are formed through sequential cleavage of the β-amyloid precursor protein (APP) by the β-amyloid precursor protein–cleaving enzyme 1 (BACE1), followed by γ-secretase. It was shown that the BACE1 levels were correlated with an increase in the oxidative stress [28]. In turn, the Aβ peptides are a source of the reactive ROS and RNS free radicals, the excessive accumulation of which leading eventually to the neuronal disintegration. Polyphenols, because of their antioxidant property, sequester the ROS and RNS, and thereby prevent the formation of toxic Aβ oligomers and modulate tau-protein hyperphosphorylation, thereby preventing the formation of the neurofibrillary tangles (NFTs) [29]. 

### 3.2. Polyphenols in the Modulation of the Signal Transduction Pathways

It was also hypothesized, through experimental evidence, that the polyphenols, including flavonoids, help prevent neuronal disintegration by interacting with major signal transduction pathways, and also, indirectly, through interaction with the blood–brain barrier and cerebral vasculature [30]. Various experimental studies have revealed a link between polyphenol consumption and improved cognitive functions, reduced risk of diabetes, hyperlipidemia, hypertension, cardiovascular diseases, and stroke [31,32]. 

**Figure 2:**
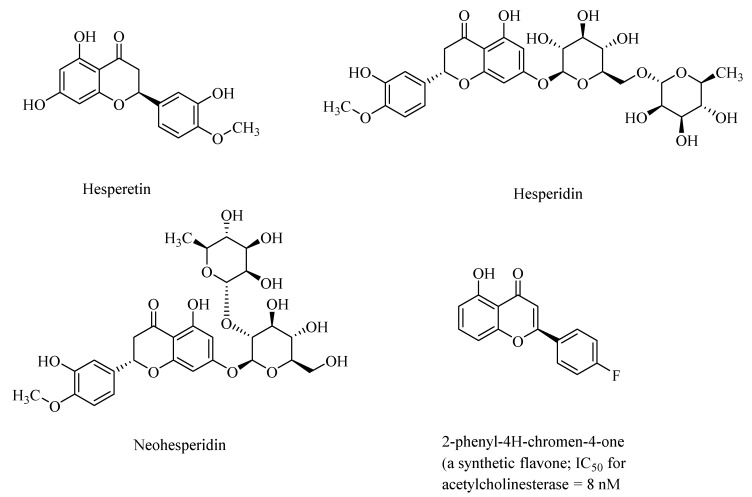
Structures of citrus flavanones, hesperitin, hesperidin, and neohesperidin and a synthetic flavone.

Synthetic analogs of 2-phenyl-4H-chromen-4-one (flavone)-derived polyphenolic compounds, such as 2-(4-fluorophenyl)-7-hydroxy-4H-chromen-4-one (Figure 2), showed high acetylcholinesterase activity, with half-maximal inhibitory constants (IC_50_) ranging from 8.0 nM to 11.8 nM) [33]. Polyphenols may also regulate NF-κB (nuclear factor-kappa β) signaling pathways through activation of the sirtuin 1 (SIRT1) proteins, and thereby attenuate neurodegeneration in AD [34].

### 3.3. Polyphenols and the Blood–Brain Barrier

Polyphenols, such as hesperetin, hesperidin, and neohesperidin, the citrus flavanones (Figure 2), are capable of crossing the blood–brain barrier, although the extent of the blood–brain barrier (BBB) permeability is widely varied among the polyphenols [35]. Accordingly, in animal studies, it was found that anthocyanins were found in the cortex and cerebellum of the rats and pigs after feeding with blueberries [36]. AD, and the accompanying dementia, is sometimes associated with cerebral amyloid angiopathy (CAA), which results from the accumulation of amyloid-β in the cerebrovasculature. Taxifolin (dihydroquercetin; Figure 1), a naturally occurring flavonoid polyphenolic compound, despite its limited blood–brain barrier, exhibited pleiotropic neuroprotective effects on cerebral amyloid angiopathy (CAA) in the mice models of AD through suppression of the amyloid-β formation, and through its anti-inflammatory effect, and thereby diminished production of the “triggering receptor expressed on myeloid cell 2” (TREM2) in the brain. The TREM2 levels are directly correlated with an increased risk of neurodegenerative diseases [37]. 

**Figure 3:**
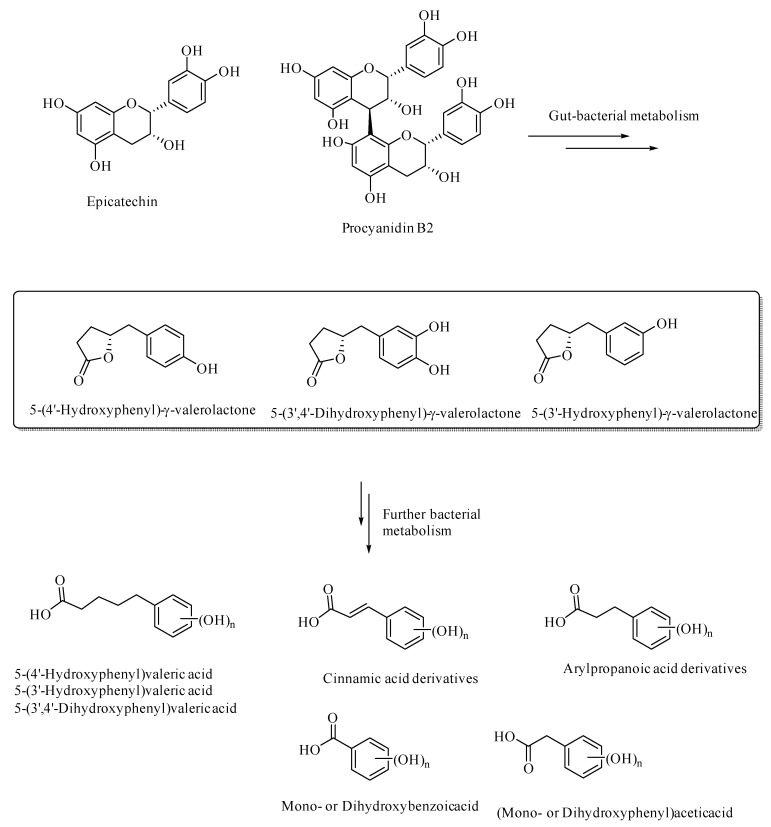
Gut bacterial metabolites of epicatechin and procyanidin B2.

Gut bacterial metabolism of the flavan-3-ols results in the formation of various aryl-γ-valerolactone and arylvaleric acid derivatives, as the major compounds, were originally shown to have protective effects against urinary tract infections, [38,39,40,41]. The aryl-γ-valerolactone metabolites also have protective effects against AD, as shown in the mouse AD models [42]. These valerolactones and their secondary metabolites were shown to selectively detoxify Aβ oligomers and thereby prevent memory loss in the mouse AD models [42]. Further metabolism of the valerolactones results in the formation of phenolic or polyphenolic degradation products, such as (hydroxyaryl)valeric acid, (hydroxyaryl)cinnamic acid, (hydroxyaryl)propanoic acid, (hydroxyaryl)acetic acid, and hydroxybenzoic acid derivatives (Figure 3). These secondary polyphenolic metabolites are relatively more bioavailable and have relatively more BBB permeability than the dietary flavanoids or flavonoids, and thereby attenuate neuroinflammation [43]. For example, 5-(hydroxyphenyl)-γ-valerolactone-O-sulfate, a secondary metabolite of the flavan-3-ols, has been demonstrated to cross the blood–brain barrier through in-silico, in-vitro, and in vivo in animal models [44]. The ellagitannin-derived polyphenols, urolithins, pyrogallol, dihydrocaffeic acid, dihydroferulic acid, and feruloylglycine, were demonstrated to be effective antiglycating and neuroprotective agents [45]. Thus, polyphenols, including those derived from gut bacterial metabolism, can be developed into effective therapeutics in various neurodegenerative diseases, as well as in diabetes. 

### 3.4. Polyphenolic Lignans in Alzheimer’s Disease

**Figure 4:**
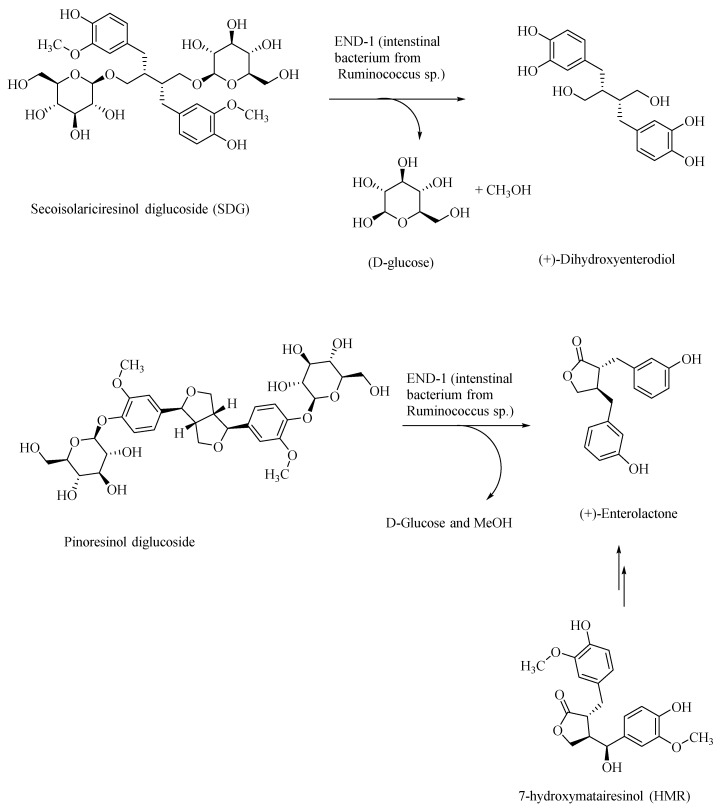
Gut bacteria in the transformation of plant-based lignans to human lignans—dihydroxyenterodiol and enterolactone.

Plant-based polyphenolic lignan compounds are abundant in flaxseeds and in other fibrous plants, such as rye, whole wheat, vegetables, and fruits. Flaxseed-derived lignans, secoisolariciresinol diglucoside (SDG) and pinoresinol diglucoside, are metabolized by the intestinal bacteria, Ruminococcus species, in humans, to give the lignans, (+)-dihydroxyenterodiol and (+)-enterolactone, respectively (Figure 4) [46]. This gut bacterial species accomplishes both deglucosylation and de-methylation of plant-based lignans to give human lignans that are of broad physiological effects. Enterolactone affords anti-cancer activity. For example, it was shown that enterolactone inhibits the growth of prostate cancer cell lines in vitro and in vivo, through a caspase-dependent pathway [47]. Enterolactone and secoisolariciresinol are inhibitors of carbonic anhydrase as well as acetylcholinesterase and butyrylcholinesterase, and thus would afford neuroprotection [48]. In AD patients, the levels of the neurotransmitter acetylcholine are substantially lowered in the neuronal synapses, leading to the memory loss. Through the inhibition of the acetylcholinesterase, these lignans afford neuroprotection and prevention of memory loss in AD patients, and thus provide an alternative treatment strategy to the use of synthetically derived acetylcholinesterase inhibitors, such as donepezil, galantamine, and rivastigmine [49,50]. The latter pharmaceuticals show only symptomatic relief, whereas lignans may, in addition, help in attenuating the progress of AD.

Dietary polyphenolic compounds also modulate the gut microbiota, and thereby affect the gut–brain axis, and thus may be used as nutraceuticals in the treatment of AD and other neurological disorders [51]. For example, the gut bacterial metabolite enterolactone, formed through intestinal bacterial metabolism of the lignan 7-hydroxymatairesinol (a constituent of coniferous tree, *Picea abies)* (HMR; Figure 4), was found to attenuate the degeneration of the striatal dopaminergic terminals in Parkinson’s disease (PD), in the PD rat models [52]. HMR, SDG, and enterolactone, through their systematic structural modifications, may afford novel neuroprotective therapeutics in AD. 

## 4. Polyphenols as Antiglycating Agents

Maillard reactions of the reducing sugars, such as D-glucose and D-ribose, with the primary amino groups of proteins (e.g., from the side chain amino groups of lysine and arginine) results in the formation of the protein crosslinks, called advanced glycation end products (AGEs). AGEs are formed when reactive aldehydes, derived from the reducing sugars, react with amines to form the Schiff bases, which upon Amadori rearrangement, followed by glycoxidations, form the AGEs. The AGEs are involved in the pathogenesis of a variety of chronic diseases, such as Alzheimer’s disease (AD), diabetes, cataract, atherosclerosis, and nephropathy [53]. Polyphenolic compounds, such as resveratrol and curcumin provide multiple mechanisms in their neuroprotection, including their role as antiglycating agents, antioxidants, and mediators of signal transduction pathways [54,55,56]. Polyphenolic compounds are effective antioxidants and free radical-trapping agents, thereby attenuating the levels of the ROS and RNS [57] and suppressing the levels of glycoxidation reactions that would otherwise lead to the formation of the toxic AGEs [53]. Thus, polyphenolic compounds attenuate the levels of AGEs at the sites of inflammation and serve as effective therapeutics for AGE-related diseases, including AD and diabetes. 

**Figure 5:**
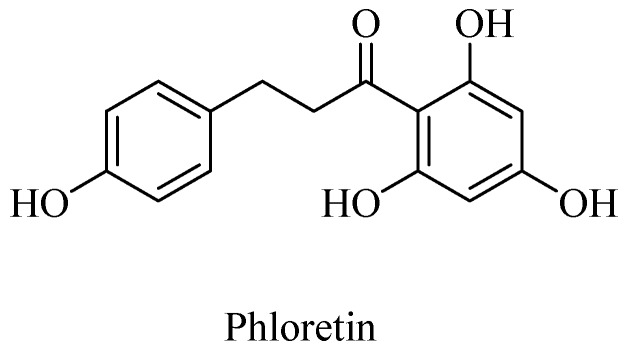
Structure of phloretin (from apple juice), an advanced glycation end product (AGE) inhibitor.

Polyphenols, such as curcumin and Vitamin E are potent antioxidants and sequester the otherwise deleterious, reactive free radicals, including ROS and RNS, and thereby afford neuroprotection in cases of AD. Curcumin along with the other AGE-inhibitor compounds, such as aminoguanidine and phenacylthiazolium compounds, potentially afford therapeutics for the neurodegenerative diseases [53,58]. A polyphenolic component in apple juice, phloretin (Figure 5), for example, acts as an AGE-inhibitor by sequestrating methylglyoxal, a reactive Maillard reaction intermediate (also formed as a byproduct of glycolysis, lipid peroxidation, and glucose auto-oxidation) that would lead to protein crosslinking, as demonstrated in the human umbilical endothelial cells (HUVECs) [59].

**Figure 6:**
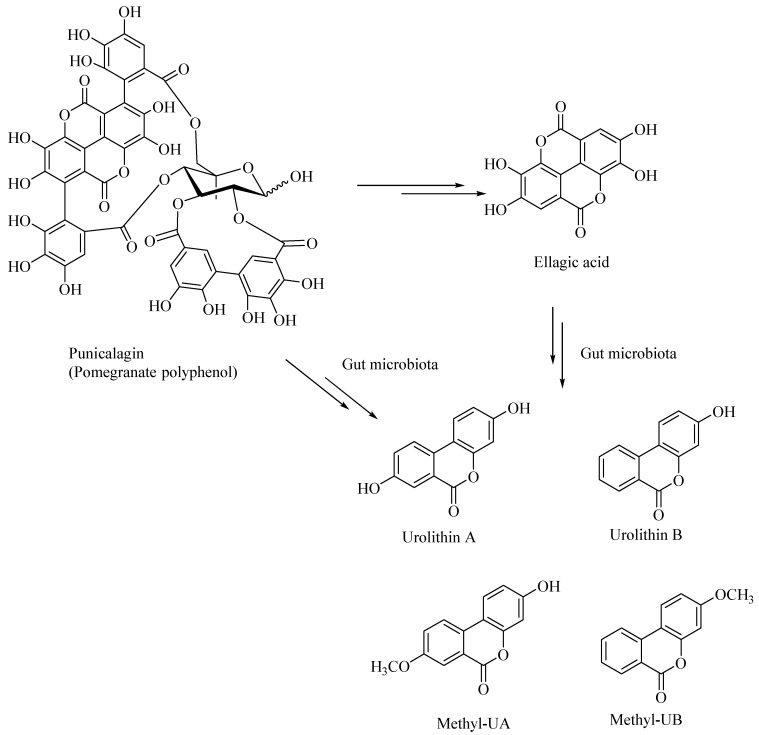
Gut bacterial metabolites of punicalagin, a pomegranate derived polyphenol.

Dietary polyphenolic compounds undergo extensive catabolism in the colon, forming a variety of small-molecule polyphenolic compounds. It was shown that the catabolites derived from the ellagitannin, such as urolithins and pyrogallol, are effective antiglycating as well as antioxidant agents [45]. Urolithins were also shown to attenuate the neuroinflammation in BV2 microglia by various other mechanisms, including the NF-κB, MAPK and Akt signaling pathways [60]. Urolithins, such as those derived from pomegranates, act as neuroprotectors and can potentially prevent the AD. The gut microbial enzymatic degradation of punicalagin, a pomegranate polyphenol, gives ellagic acid (a polyphenolic bis-lactone), and Urolithin-A, Urolithin-B, Methyl-UA, Methyl-UB (Figure 6). These pomegranate-derived urolithins, such as Urolithin-A and Urolithin-B, were shown to attenuate neuroinflammation in the BV-2 microglia and human SH-SY5Y neuronal cells [61]. The latter urolithins, prevented β-amyloid fibrillation in vitro, whereas the pomegranate extracts, including the ellagitannins, did not show any neuroprotective effects [61].

Chlorogenic acid-derived catabolites, such as dihydrocaffeic acid, dihydroferulic acid and feruloylglycine, were also shown to be effective neuroprotectors [45]. Polyphenols also affect the gut microbiota composition, thereby modulating the composition of the metabolites [62]. Thus, the effect of polyphenolic compounds on neurodegeneration is mediated by multiple mechanisms, among which the antiglycating and antioxidant activity of the polyphenols may play a significant role in neuroprotection in AD and other neurodegenerative diseases.

## 5. Conclusions and Outlook

Dietary polyphenolic compounds (plant lignans) are metabolized by the gut microbiota to numerous small-molecule phenolic compounds, called human lignans, and a variety of these polyphenolic compounds protect against human pathologies, including AD. Notably, some of these polyphenolic compounds have favorable blood–brain penetration properties. Polyphenolic compounds exhibit antiglycating and antioxidant properties and also modulate the signal transduction pathways in the neuronal cells. Because of these favorable properties, polyphenols or their synthetic analogs can potentially serve as nontoxic therapeutic candidates in the prevention and management of AD and other neurological disorders. Furthermore, polyphenolic compounds, such as curcumin, can be incorporated into polymeric nanoparticles—for example, those based on poly(ethylene glycol) (PEG), poly(lactic acid) (PLA), poly(ethyleneimine) (PEI), poly(lactic-co-glycolic acid) (PLGA), chitosan, and poly(vinylpyrrolidone) (PVP), to enhance their blood–brain barrier (BBB) permeability [63]. Curcumin–PEG–PLA nanoparticles, for example, cross the BBB six-fold more effectively as compared to the curcumin alone [63,64]. Thus, gut bacterial metabolism-derived polyphenolic compounds, in nanoparticulate form, may prove to be effective therapeutics for AD and other neurological disorders, such as TBI and PD.
